# Systemic Sarcoidosis Unmasked by Cushing's Disease Surgical Treatment

**DOI:** 10.1155/2016/6405840

**Published:** 2016-07-25

**Authors:** Daniele Bongetta, Cesare Zoia, Francesco Lombardi, Elisabetta Lovati, Pietro Lucotti, Paolo Gaetani

**Affiliations:** ^1^Department of Clinical-Surgical, Diagnostic and Pediatric Sciences, Università degli Studi di Pavia, Viale Brambilla 74, 27100 Pavia, Italy; ^2^Neurosurgery Unit, Fondazione IRCCS Policlinico S. Matteo, Viale Golgi 19, 27100 Pavia, Italy; ^3^Endocrinology Unit, Internal Medicine I, Fondazione IRCCS Policlinico S. Matteo, Viale Golgi 19, 27100 Pavia, Italy

## Abstract

Diseases responsive to glucocorticoids, like sarcoidosis, are rarely masked by Cushing's syndrome. An ACTH secreting pituitary adenoma is a possible cause of Cushing's syndrome and its resection can make a subclinical sarcoidosis clear. Only few cases of sarcoidosis following the treatment of hypercortisolism are reported in literature. We report a case of sarcoidosis after the resection of an ACTH secreting pituitary adenoma.

## 1. Introduction

Follow-up evaluations of surgically operated pituitary adenoma patients are often staged multidisciplinarily between different specialists. We would like to share here a case of an unusual complication following transsphenoidal surgery for Cushing's disease that may arise at midlong follow-up timing to warn all the pituitary-related specialists.

## 2. Case Report

**Figure 1:**
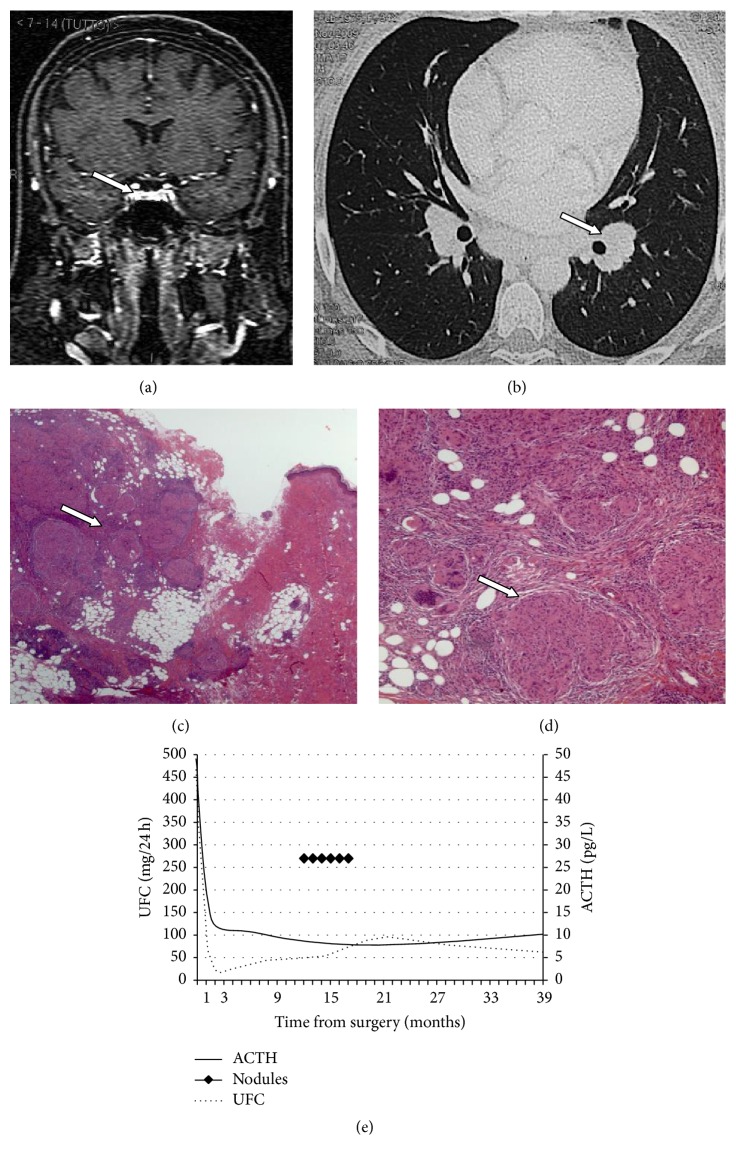
(a) Coronal MRI scan showing the pituitary microadenoma. (b) CT scan: multiple enlarged lymph nodes in the hilar-mediastinal region bilaterally. (c) 25x magnification: hematoxylin and eosin. Presence in the deep dermis and hypodermis of numerous epithelioid granulomas. (d) 100x magnification: hematoxylin and eosin. (e) Urinary free cortisol (UFC) and adrenocorticotropic hormone (ACTH) levels trend. Sarcoidosis nodules were present approximately from the 12th to the 17th month after surgery.

**Figure 2:**
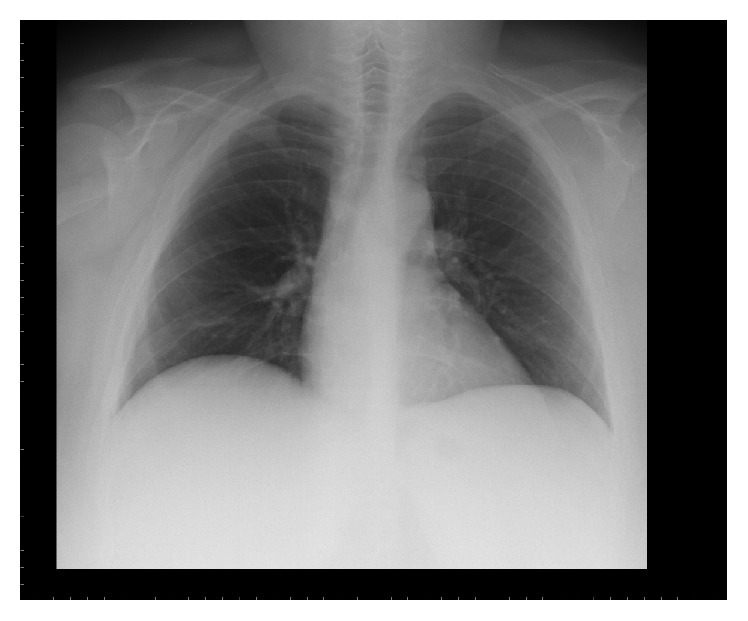
Preoperative chest X-ray.

A 33-year-old woman had a 10-month history of amenorrhea and asthenia and weight gain (15 kgs). On physical examination, the patient was severely obese (127 kg), with predominantly central disposal of fat, abdominal purple striae, hirsutism, and dorsal hump. Blood tests revealed high levels of cortisol (1510 *μ*g/dL): basal and dynamic pituitary function evaluation showed a normal value of ACTH with discrete response to stimulation with DDAVP, elevated cortisol with failure to respond to the CRH test stimulus, elevated urinary free cortisol, PTH elevated with normal serum calcium, and deficient values of gonadotropins and ovarian hormones. An MRI study completed with thin section dynamic contrast-enhanced sequences showed the presence of a pituitary microadenoma (Figure 1(a)). No other alterations were evident at preoperative imaging studies (Figure 2). A diagnosis of Cushing's disease was posed and she was subjected to endoscopic transsphenoidal surgery. The postoperative course was characterized by transient diabetes insipidus and rib pain caused by multiple osteoporosis-related rib fractures. She was discharged on the fifth postoperative day. First-year follow-up endocrinological evaluations were at 1, 5, 8, and 12 months. Yet after the first month, serum levels of ACTH and urinary free cortisol were to the lower limits of standard with good response to the stimulus testing; no other major pituitary malfunctions were present. Cortisol replacement was continued and progressively tapered during the follow-up. One year after intervention, though, she noticed the presence of painless, firm, subcutaneous nodules on both hands and elbows and on the left knee, associated with asthenia and unspecific malaise. Body weight was diminished (−13 kgs), diabetes was under good control, and there were no concomitant infectious diseases. A biopsy of one of the nodules was performed and histological examination showed nonnecrotizing epithelioid cell granuloma, consistent with the diagnosis of sarcoidosis (Figures 1(c) and 1(d)). She was then subjected to an X-ray study, finding an enlargement of the hilar shadows on both sides. A CT scan with contrast medium showed the presence of multiple enlarged lymph nodes in the hilar-mediastinal region bilaterally, with a maximum diameter in subcarinal area of approximately 4 cm (Figure 1(b)). There were no pleural or parenchymal lesions with characters of activity or signs of interstitial disease. Pulmonary function tests were normal. QuantiFERON-TB Gold analysis,* M. tuberculosis* complex PCR amplification test, and screening blood tests for autoimmune diseases were negative, while serum ACE was 40.1 Ui/L (8–52). Cholecalciferol supplementation has been provided to the patient on a monthly basis to correct deficient 25-hydroxy vitamin D level found before surgery. Subsequent PTH and calcium levels were interpreted as secondary to underlying sarcoid. At skin nodules first-time appearance, the patient was still on low doses of daily steroid therapy (cortisone acetate 12.5 mg/day). Cortisone acetate therapy was then suggested until its permanent discontinuation 5 months later (Table 1). Skin nodules progressively regressed in five months. Radiological and endocrinological follow-up at 5 years showed no signs of disease progression or recurrence for both sarcoidosis and Cushing's disease; no therapy is prescribed and body weight dropped a total of 25 kgs.

## 3. Discussion

Corticoids are a common treatment option for many immune-related diseases. New onset or worsening of many of these diseases after Cushing syndrome remission has been reported in the literature including arthritis, vasculitis, dermatitis, celiac disease, and systemic lupus erythematosus [1]. Sarcoidosis is a systemic disorder of unknown etiology whose treatment is often successful with continuous glucocorticoid prescription. To date, we found only five other previous reports of sarcoidosis after Cushing's disease surgical treatment [1–5]. All of the cases (4 females, 2 males) had skin and lung involvement while three cases had also mediastinal masses and one had a localisation in the eye. Since mean delay to onset of symptoms is 14.5 months (3–48), a strict follow-up and widespread knowledge of this potentially harmful condition in both endocrinological and neurosurgical communities are to be recommended.

## Figures and Tables

**Table 1:** Calcium, PTH, and 25-OH vitamin D levels and cortisone acetate daily therapy throughout three years after intervention.

	1 month	5 months	8 months	12 months	18 months	24 months	36 months
Calcium (mg/dL, normal range 8.5–11.0)	9.2	8.9	8.9	10.7	9.8	10.1	9.3
Intact parathyroid hormone (pg/mL, normal range 12.0–72.0)	114	47.4	—	15.4	55.3	—	43.7
25-Hydroxy vitamin D (ng/mL)	—	27.7	—	30.1	—	34.8	—
Cortisone acetate (mg/day)	25	12.5	12.5	12.5	/	/	/
